# Bridges and boundaries in dementia with lewy bodies: Heterogeneity of clinical and neuroimaging features

**DOI:** 10.1002/alz.095103

**Published:** 2025-01-09

**Authors:** Achcha Chimagomedova, Oleg Levin

**Affiliations:** ^1^ Russian Medical Academy of Continuous Professional Education, Moscow, Moscow Russian Federation

## Abstract

**Background:**

White matter hyperintensity (WMH), which is considered a sign of vascular lesions, is often found in patients with DLB, but its association with vascular lesions has not been proven. We assessed WMH in patients with DLB and markers of vascular damage and vascular risk factors.

**Method:**

We examined 78 patients that fulfilled clinical criteria of DLB. The clinical assessment included MoCA, MMSE, ACE‐R, IQCODE, NPI‐4, UM‐PDHQ, UPDRS‐III, RBD1Q, orthostatic hypotension test. All patients underwent 1,5 T brain MRI. WMH was evaluated with Fazekas score and ARWMC. Patients were performed ultrasound assessment of flow‐mediated vasodilation. Laboratory investigation included Willebrand factor, lipid profile, microalbumin/creatinine ratio.

**Result:**

MRI revealed with moderate to severe WMH in 43 participants. There were no significant differences in vascular risk factors, vessel reactivity, laboratory results between patients with or without WMH. These findings were associated more likely with hyppocampal atrophy and disease duration than vascular risk factors.

**Conclusion:**

WMH in DLB may be associated with neurodegenerative pathology. Further investigations with diffusion MRI techniques and prospective studies can be helpful in interpretation of neuroimaging findings and understanding of its contribution to the course of the disease.

